# OCT4-mediated upregulation of DUSP6 promotes metastasis in non-small-cell lung cancer

**DOI:** 10.7150/jca.108663

**Published:** 2025-10-01

**Authors:** Bing-Hua Su, Chung-Teng Wang, Chia-Sing Lu, Tang-Hsiu Huang, Tzu-Chun Wu, Yu-Chu Su, Yu-Chih Wu, Yi-Ting Yen, Yau-Lin Tseng, Li-Hsin Cheng, Chi-Won Suk, Ai-Li Shiau, Jia-Ming Chang, Chao-Liang Wu

**Affiliations:** 1 School of Respiratory Therapy, College of Medicine, Taipei Medical University, Taipei, Taiwan.; 2 TMU Research Center of Thoracic Medicine, Taipei Medical University, Taipei, Taiwan.; 3 Department of Microbiology and Immunology, College of Medicine, National Cheng Kung University, Tainan, Taiwan.; 4 Tong Yuan Diabetes Center, College of Medicine, National Cheng Kung University, Tainan 70101, Taiwan.; 5 Department of Biochemistry and Molecular Biology, College of Medicine, National Cheng Kung University, Tainan, Taiwan.; 6 Division of Chest Medicine, Department of Internal Medicine, National Cheng Kung University Hospital, College of Medicine, National Cheng Kung University, Tainan, Taiwan.; 7 Division of Thoracic Surgery, Department of Surgery, National Cheng Kung University Hospital, College of Medicine, National Cheng Kung University, Tainan, Taiwan.; 8 Core Laboratory of Organoids Technology, Office of R&D, Taipei Medical University, Taiwan.; 9 Division of Pulmonary Medicine, Department of Internal Medicine, Wan Fang Hospital, Taipei Medical University, Taipei, Taiwan.; 10 Thoracic Division, Department of Surgery, Ditmanson Medical Foundation Chiayi Christian Hospital, Chiayi, Taiwan.; 11 Institute of Molecular Biology, National Chung Cheng University, Chiayi, Taiwan.; 12 Ditmanson Medical Foundation Chia-Yi Christian Hospital, Chiayi, Taiwan.

**Keywords:** octamer-binding transcription factor 4 (OCT4), dual-specificity phosphatase 6 (DUSP6), metastasis, non-small-cell lung cancer (NSCLC)

## Abstract

The roles of cancer stem cells and Octamer-binding transcription factor 4 (OCT4) have been implicated in human tumorigenesis and metastasis. However, the role of OCT4 in the metastasis of non-small-cell lung cancer (NSCLC) remains undetermined, especially regarding stem cell-related pathways. Previous research has reported that dual-specificity phosphatase 6 (DUSP6), a mitogen-activated protein kinase (MAPK) phosphatase, is associated with cancer cells that display anti-apoptotic, migratory, and drug-resistance phenotypes. However, the regulation of DUSP6 in NSCLC is unclear. This study focused on the role of OCT4 in NSCLC, particularly its interaction with DUSP6. Here, we show a positive correlation between OCT4 and DUSP6 expression in NSCLC cells. Overexpression of OCT4 increased, whereas knockdown of OCT4 reduced DUSP6 expression. Luciferase reporter and chromatin immunoprecipitation (ChIP) assays revealed that OCT4 transactivated DUSP6 expression by directly binding to the DUSP6 promoter, indicating that DUSP6 is a downstream target of OCT4. Furthermore, knockdown of DUSP6 in OCT4-overexpressing A549 human NSCLC cells decreased cell migration *in vitro* and reduced tumor growth and pulmonary metastasis in NOD/SCID mice. In conclusion, our findings highlight the importance of the OCT4-DUSP6 pathway in NSCLC progression. Furthermore, the OCT4-DUSP6 axis is a potential therapeutic target for NSCLC.

## Introduction

Lung cancer remains the foremost cause of cancer-related deaths globally 1. In 2023, it represented 21% of all cancer fatalities in the United States. Non-small-cell lung cancer (NSCLC), often inoperable, poses a significant challenge with a survival rate of approximately 23% within five years. The invasion and metastasis of NSCLC are the main causes of cancer death and treatment failure. Emerging evidence supports that tumor pathogenesis is induced by cancer stem cells (CSCs) 2. The cancer stem cell hypothesis suggests that malignant tumors are initiated and maintained by progenitor cells within the tumor that exhibit biological characteristics of normal adult stem cells 3. Consequently, identification of effective molecular targets for NSCLC treatment is crucial.

Dual-specificity phosphatase 6 (DUSP6), a member of the DUSP family, removes phosphate from tyrosine and serine/threonine residues of substrates 4. It negatively regulates mitogen-activated protein kinase (MAPK) activity by dephosphorylating the essential threonine and tyrosine residues in the activation loop 5, 6. As a MAPK phosphatase family member, DUSP6 inactivates extracellular signal-regulated kinase 2 (ERK2), impacting both tyrosine and serine/threonine residues. In NSCLC, DUSP6 has emerged as a high-risk gene signature associated with relapse and reduced overall survival 7. Its overexpression in NSCLC correlates with poor prognosis and is prevalent in the advanced stages of the disease. However, the mechanisms underlying DUSP6 upregulation in NSCLC remain unclear. In addition to lung cancer, DUSP6 influences other cancers, including its role in invasiveness and migration in papillary thyroid cancer 8, 9 and in promoting the growth of glioblastoma 10. Increased DUSP6 expression is associated with cancer cells that display anti-apoptotic, migratory, and drug-resistance phenotypes 10. Silencing of DUSP6 significantly decreases the cell viability and migration rate of FRO thyroid cancer cells 8, suggesting that DUSP6 plays a key role in malignant tumor metastasis.

Octamer-binding transcription factor 4 (OCT4), part of the POU homeobox gene family 11, is recognized as a critical regulator of embryonic stem cell pluripotency 12. Its dysregulated expression in various solid tumors is linked to cancer progression. OCT4 proportionally increases tumorigenesis risk, and its elevation correlates with increased motility and transmigration of cancer cells in melanoma 13 and glioblastoma 14. In human NSCLC 15, 16, OCT4 is predominantly expressed in tumor tissues, with higher levels indicating poorer prognosis. When combined with Nanog, OCT4 significantly enhances lung adenocarcinoma malignancy, influencing motility by upregulating genes, such as Snail and Slug 17. Our previous research in bladder cancer aligns with these findings, where OCT4 is associated with worse outcomes and increased metastasis, promoting cancer cell migration through the upregulation of specific growth factors and matrix metalloproteinases 18.

Despite these insights, the exact mechanisms underlying the contribution of OCT4 to poor prognosis and metastatic potential remain elusive. Previous array data showed that knocking down OCT4 in mouse embryonic stem cells significantly decreased DUSP6 expression 19. We further identified three OCT4 binding sites within the DUSP6 promoter. These findings suggest that OCT4 may transcriptionally regulate DUSP6 expression. In the present study, we explored the association between OCT4 and DUSP6 expression in NSCLC and investigated whether OCT4 exacerbated tumor malignancy and metastasis by directly upregulating DUSP6 expression. Understanding the OCT4/DUSP6 pathway may open new avenues for therapeutic interventions in NSCLC.

## Materials and Methods

### Clinical specimens and cell lines

For clinical analysis, formalin-fixed, paraffin-embedded sections of human lung tumor tissues and adjacent non-tumor parts were collected from patients who underwent surgery at NCKU Hospital, Tainan, Taiwan. All procedures followed the Declaration of Helsinki, with informed consent obtained from all participants. The study was approved by the Institutional Review Board of NCKU Hospital (IRB No. B-ER-105-406). Human lung cancer cell lines A549 (ATCC CCL-185), H1299 (ATCC-CRL-5803), and H661 (ATCC-HTB-183TM) were purchased from the American Type Culture Collection (ATCC, Manassas, VA, USA). In addition, CL1-0, CL1-1, CL1-5, and CL1-5-F4 cell lines were kindly provided from Pan-Chyr Yang (Department of Internal Medicine, National Taiwan University Hospital, Taiwan) 20. Except for the large cell lung cancer cell line (H661), all human lung cancer cell lines belong to NSCLC. All cancer cells were cultured in DMEM (Gibco, Invitrogen, Carlsbad, CA, USA) supplemented with 10% cosmic calf serum (Hyclone, Logan, UT, USA), 2 mM L-glutamine, and 50 μg/ml of gentamicin at 37 ℃ in a 5% CO_2_ environment. A549 and cells were transduced with lentiviral vectors encoding OCT4 or luciferase, followed by puromycin selection. H1299 cells were transfected with an OCT4 expression plasmid or a control plasmid by PEI, followed by G418 selection.

### Plasmids

The lentiviral vector encoding human OCT4 (pSin-EF2-OCT4-Pur, Addgene plasmid 16579) was obtained from Addgene (http://www.addgene.org). Moreover, the coding region of OCT4 was removed from pSin-EF2-OCT4-Pur by *Spe*I and *Eco*RI digestion, and the resulting large fragment was treated with the Klenow fragment of *E. coli* DNA polymerase I and self-ligated using T4 DNA ligase to generate the lentiviral vector pSin-EF2-Pur encoding no transgenes 21. To create a control lentiviral vector encoding GFP, the GFP cDNA fragment was excised from pEGFP-N1 (Clonetech, Palo Alto, CA, USA) by digestion with *Nhe*I and *Mfe*I and then cloned into the *Spe*I/*Eco*RI sites of pSin-EF2-OCT4-Pur to replace OCT4, designated pSin-EF2-GFP-Pur 22. In addition, the cDNA fragment of human OCT4 was obtained from pSin-EF2-OCT4-Pur by PCR amplification and cloned into a TA vector. The resulting PCR amplicons were digested with *Bgl*II and *Sal*I and subcloned into the *Bam*HI/*Sal*I sites of pCMV-tag2B (Stratagene, La Jolla, CA, USA) to generate a Flag-tagged OCT4 expression plasmid (pCMV-tag2B-hOCT4). For shRNA-based knockdown experiments, pLKO.1-puro-based lentiviral vectors including stem-loop cassettes encoding shRNA for human OCT4 (TRCN0000004880 and TRCN0000004883, designated shOCT4 #2 and shOCT4 #5), human DUSP6 (TRCN0000010707 and TRCN0000233474, designated shDUSP6 #2 and shDUSP6 #4), human NOTCH1 (TRCN0000350253) and luciferase (TRCN0000072246, designated shLuc) were obtained from the National RNAi Core Facility, Taiwan. Various recombinant lentiviruses were produced as previously described 21, 23.

### Immunoblotting

For immunoblotting, 30 μg of cell lysates were separated on 10% SDS-polyacrylamide electrophoresis gels and transferred to PVDF membranes (Millipore, Bedford, MA, USA). The membranes were then incubated with rabbit anti-human OCT4 antibody (#2750, 1:1000, Cell Signaling, Beverly, MA, USA), rabbit anti-human DUSP6 antibody (#3058, 1:1000, Cell Signaling), rabbit anti-MMP-2 antibody (ab97779, 1:1000, Abcam, Cambridge, UK), rabbit anti-MMP-9 antibody (ab137867, 1:1000, abcam), or rabbit anti-NICD antibody (#4147 1:1000, Cell Signaling). Antibody-protein complexes were detected with horseradish peroxidase (HRP)-conjugated goat anti-rabbit IgG (1:10,000, Jackson Immuno Research, West Grove, PA, USA) and visualized using an enhanced chemiluminescence (ECL) kit (Amersham, GE Healthcare, Buckinghamshire, UK). Subsequently, β-actin was detected with mouse monoclonal anti-β-actin-peroxidase antibody (A3854, Sigma-Aldrich, St. Luis, MO, USA) on the same membrane to serve as a loading control.

### Immunohistochemistry (IHC) staining

Formalin-fixed, paraffin-embedded (FFPE) human lung tumor tissues and adjacent non-tumor tissues were cut into 4-μm sections. Slides were first deparaffinized in xylene (2 × 10 min) and rehydrated through a graded ethanol series (100%, 95%, 70%, and distilled water). Antigen retrieval was performed by incubating the slides with proteinase K (20 μg/mL in PBS) at room temperature for 10 minutes. Endogenous peroxidase activity was blocked using 3% hydrogen peroxide in methanol for 10 minutes. After washing with PBS, non-specific binding was blocked by incubating the sections with 10% bovine serum albumin (BSA) in PBS for 30 minutes at room temperature. The slides were then incubated overnight at 4°C with the following primary antibodies diluted in PBS with 1% BSA: mouse monoclonal anti-OCT4 (sc-5279, Santa Cruz Biotechnology, Santa Cruz, CA, USA) or rabbit anti-DUSP6 antibody (sc-28902, Santa Cruz Biotechnology). After washing, the sections were incubated with appropriate horseradish peroxidase (HRP)-conjugated secondary antibodies (anti-mouse or anti-rabbit, The Jackson Laboratory, Sacramento, CA, USA) for 120 minutes at room temperature. Antibody binding was visualized using 3-amino-9-ethylcarbazole (AEC, Zymed, San Francisco, CA, USA) as the chromogenic substrate. Slides were then counterstained with hematoxylin, dehydrated, and mounted.

### Reverse transcription-polymerase chain reaction (RT-PCR) and Real-time quantitative RT-PCR (qPCR)

Total RNA was extracted using Trizol (Invitrogen) reagent according to the manufacturer's protocol. Total RNA (2 μg) was reverse-transcribed into cDNA using a Verso™ cDNA synthesis kit (Thermo Fisher Scientific, Waltham, MA, USA). For RT-PCR, the primers used included human OCT4, 5'-GTCCGAGTGTGGTTCTGTA-3' (sense) and 5'-CTCAGTTTGAATGCATGGGA (antisense); human DUSP6, 5'-CAAAGGGAGAAAGAGCAGTATGCC-3' (sense) and 5'-CAAAAGTATTGCATTTGAGGTGACAC-3' (antisense); and human glyceraldehyde-3-phosphate dehydrogenase (GAPDH), 5'-ACTTCAACAGCGACACCCACT-3' (sense) and 5'-GCCAAATTCGTTGTCATACCAG-3' (antisense). PCR was performed with an automated thermocycler (Biometra, Göttingen, Germany) according to standard protocols. The PCR products were separated by 1% agarose gel electrophoresis. For qPCR, the following primers were used: human OCT4, 5'-CCTGAAGCAGAAGAGGATCACC-3' (forward) and 5'-AAAGCGGCAGATGGTCGTTTGG-3' (reverse); human DUSP6, 5'-AGCTCAATCTGTCGATGAACG-3' (forward) and 5'-GCGTCCTCTCGAAGTCCAG-3' (reverse); human GAPDH, 5'-ACAACTTTGGTATCGTGGAAGG-3' (forward) and 5'-GCCATCACGCCACAGTTTC-3' (reverse). The cDNA and primers were mixed with the SYBR premix Ex Taq (Takara Bio USA, Inc). The qPCR was performed with the MyGo Pro® RealTime PCR System (Novacyt, Camberley, Surrey, UK) according to standard protocols. The relative mRNA expression of different genes was determined using the 2-ΔΔCT method, with the value obtained by subtracting the Ct value for GAPDH mRNA from the Ct value for different mRNA species.

### Reporter plasmid construction and luciferase assay

To generate series-deleted human DUSP6 promoter reporter vectors, PCR was performed using primers corresponding to sequences -1829 bp to -1 bp, -713 bp to -1 bp, -465 bp to -1 bp, and -239 bp to -1 bp, with genomic DNA from A549 cells as a template. A site-directed mutagenesis of the mutant DUSP6 promoter construct in which the putative OCT4-binding site (-423 to -416, wild-type sequence ATGCTAAT) was altered to ATGCTAGG. This mutation disrupts the core consensus sequence recognized by the OCT4 POU domain and was used to assess the functional relevance of this element in promoter activation assays. The site-directed mutagenesis approach was introduced according to the manufacturer's instructions (QuikChange Lightning Site-Directed Mutagenesis Kit, Agilent, CA, USA). The PCR fragments were cloned into a TA vector and sequenced. The plasmids were then cut, and the released DUSP6 promoter fragments were cloned into the single dual-luciferase reporter vector pFRL2 to generate pFRL2-hDUSP6p-Luc 24. For transient transfections, 5 × 10^4^ of A549, H1299, and CL1-5 cells in 24-well plates were transfected with a total of 1 μg of plasmid DNA using Lipofectamine 2000 or PEI 25,000 (0.45 mg/ml, pH 7.0) according to the manufacturers' instructions. Cell lysates were harvested 48 h after transfection, and their firefly and *Renilla* luciferase activities were determined using a dual-light luciferase reporter assay system (Promega, Madison, WI, USA). The ratio of firefly luciferase activity to Renilla luciferase activity was expressed as relative light units (RLU).

### Chromatin immunoprecipitation (ChIP) assay

A ChIP assay was conducted using the EZ-ChIP (Millipore) following the manufacturer's instructions. H1299 cells (1 × 10^7^) in 15-cm culture dishes were cross-linked in 1% formaldehyde solution for 10 min at 37 ℃. After being washed with PBS, cells were harvested in 2 ml of PBS and then pelleted by centrifugation. The pellets were resuspended in 1 ml of the ChIP kit SDS lysis buffer containing protease inhibitor cocktail. Samples (400 μl) were sonicated to shear the DNA to an average length of approximately 500 base pairs. Samples were centrifuged, and the supernatants were diluted in a 2-ml tube with the ChIP kit dilution buffer. Subsequently, samples (200 μl) were supplemented with 800 μl dilution buffer and 60 μl protein G agarose beads, followed by rotation at 4 ℃ for 1 h. After centrifugation, the supernatant was transferred to a new Eppendorf tube. Each OCT4 immunoprecipitation (300 μl) was carried out with 6 µg of mouse monoclonal anti-human OCT4 antibody (sc-5279, Santa Cruz). Immunoprecipitation (300 μl) with purified normal mouse IgG was used as a non-specific precipitator and negative control. After overnight immunoprecipitation on a rotator at 4 ℃, samples were washed, eluted, and DNA was reverse-crosslinked with 5 M NaCl at 65 ℃ for 5 h. DNA was purified, and the final DNA pellets were eluted in double-distilled H_2_O and subjected to PCR with specific primers to amplify the DUSP6 promoter region containing OCT4 binding sites.

### Cell invasion assay

Cell invasion was analyzed using Boyden chamber assays with 8-μm pore polycarbonate filters (Neuro Probe, Gaithersburg, MD, USA) coated with 0.1 μg/ml of gelatin (Sigma-Aldrich). A549 cells were incubated with serum-free DMEM for 4 h and then transferred to the chamber. The lower chambers, containing 28 μl of complete medium, were covered with the filters. Various derivatives of A549 cells (5 × 10^5^/well), including A549/OCT4, A549/Vector, A549/OCT4_shDUSP6, and A549/OCT4_shLuc cells, were seeded in the upper chambers in serum-free medium. For the A549/shLuc and A549/shOCT4, to minimize the confounding effect of cell proliferation, both cell lines were treated with 5 μg/mL mitomycin C in serum-free medium in the upper chambers. After 4 h of incubation at 37 ℃, all the cells were fixed with methanol and stained with Giemsa solution (Invitrogen) for 1 h. Cells on the upper surface of the filter were scraped off with cotton buds. Invaded cells on the underside of the filter were then photographed and counted using phase-contrast microscopy.

### Animal studies

Male 7- to 9-week-old NOD/SCID mice were purchased from the Laboratory Animal Center of NCKU. The experimental protocol adhered to the rules of the Animal Protection Act of Taiwan and was approved by the Laboratory Animal Care and Use Committee of NCKU. Groups of mice were subcutaneously inoculated with 1 × 10^6^ of A549/OCT4 or A549/Vector cells. In another set of the experiment, groups of mice were subcutaneously inoculated with 1 × 10^6^ of A549/OCT4, A549/OCT4_shDUSP6, or A549/OCT4_shLuc cells. Palpable tumors were measured at different time points in two perpendicular axes with a tissue caliper, and the tumor volume was calculated using the formula: (length of tumor) × (width of tumor)^2^ × 0.45. The mean tumor volumes were calculated when all the mice within the same group were alive. Subsequently, tumor-bearing mice were sacrificed to detect and quantify metastatic nodules in the lung. Histochemistry and immunohistochemistry were also performed to detect tumor nodules as well as OCT4 and DUSP6 expression in the lung. For A549/OCT4 or A549/Vector cells, serial measurements of tumor volume continued until day 120, which was prospectively designated the primary tumor growth endpoint because (i) intergroup differences were reliably detectable by days 100-120, and (ii) tumors in several mice approached the pre‑approved humane size limit (~1.5 cm maximum dimension). To minimize distress and avoid confounding from necrosis in larger lesions, routine caliper measurements were discontinued after day 120. Mice were observed without further caliper measurements until day 156, a timepoint chosen based on a preliminary experiment establishing that macroscopic lung metastases from subcutaneous A549 tumors in NOD/SCID mice become consistently detectable only after 150 days. Under close welfare monitoring, at day 156, mice were euthanized, lungs harvested, and metastatic nodules enumerated under a dissecting microscope.

### Statistical analysis

Data are expressed as mean ± standard error of the mean (SEM). Statistical differences were compared by Student's *t*-test between two groups and by one-way ANOVA with Bonferroni post hoc test among three or more groups. Correlations were analyzed using Pearson's correlation coefficient. Differences in the tumor volume between groups were compared by repeated-measures analysis of variance (ANOVA) using SAS software version 9.1 (GLM program; SAS Institute, Cary, NC, USA). Any p-value of < 0.05 was regarded as statistically significant.

## Results

### Expression of OCT4 and DUSP6 is positively correlated in lung cancer cells

**Figure 1:**
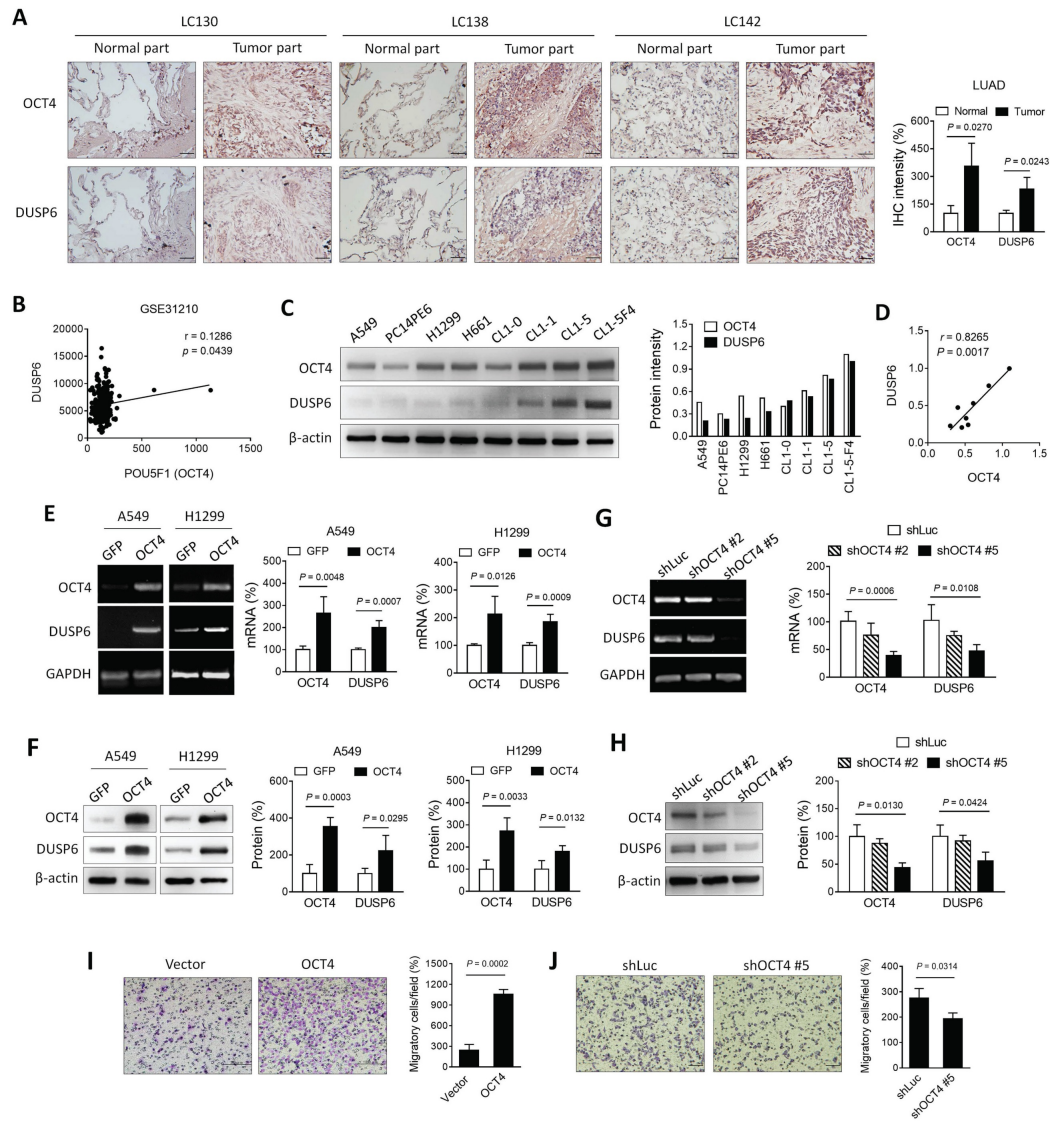
** Expression of OCT4 and DUSP6 is positively correlated in lung adenocarcinoma (LUAD) cells. (A)** Immunohistochemical (IHC) staining and quantification of paraffin-embedded tumor parts and normal tissue sections for OCT4 and DUSP6 expression (× 200 magnification, scale bar = 50 μm). IHC intensity was quantified using the ImageJ software. Values shown are levels of immunointensity in individual specimens, with the mean level in normal tissue arbitrarily set to 100, in three randomly selected fields in each section (n=3). **(B)** A positive correlation between the expression of POU5F1 (OCT4) and DUSP6 in the expression profiles of 226 pathological stage I-II lung adenocarcinomas (NCBI Gene Expression Omnibus: GSE31210). The correlation was assessed using Pearson's correlation coefficient (*r*=0.1268, *P*=0.0439). **(C)** Immunoblot analysis and quantification of endogenous expression levels of OCT4 and DUSP6 in eight lung cancer cell lines. Expression of β-actin served as the loading control. **(D)** Positive correlation between OCT4 and DUSP6 expression levels in cancer cell lines. Band intensity was quantified using the ImageJ software, and correlations were assessed using Pearson's correlation coefficient (*r=*0.8265*, P=*0.0017).** (E, F)** Increased expression of DUSP6 mRNA (E) and protein (F) in lung cancer cells overexpressing OCT4 as examined by RT-PCR (E, *left*), quantitative real-time PCR (qPCR) (E, *right*), and immunoblotting (F). A549 and H1299 cells were transduced with lentiviral vectors encoding OCT4 or luciferase, followed by puromycin selection. Expression of GAPDH (E) and β-actin (F) served as the internal control. **(G, H)** Knockdown of OCT4 by OCT4 shRNA reduces DUSP6 expression. A549 cells were transduced with lentiviral vectors expressing shRNAs specific to OCT4 (shOCT4 #2 and shOCT4 #5) or to luciferase (shLuc). Levels of OCT4 and DUSP6 mRNA and protein were examined by RT-PCR (G, *left*), qPCR (G, *right*), and immunoblotting (H). **(I, J)** Enhanced migratory and invasive capability of OCT4-overexpressing A549 cells, or reduced that of OCT4-knockdown A549 cells. Migration of A549/OCT4 and A549/Vector cells or A549/shLuc and A549/shOCT4#5 cells was detected by the Boyden chamber assay. Cells that migrated through the membrane of the lower surface in the Boyden chamber were stained with Giemsa solution and visualized by light microscopy (× 200 magnification, scale bar = 200 μm) (I, J, *left*) and quantified (I, J, *right*). Migratory cells in three fields in each membrane were counted. All quantification values and error bars of (A) and (E-J) shown were mean ± SEM. SEM, standard error of the mean.

As a transcription factor, OCT4 exerts its regulatory function by binding to the OCT4 response element (ORE), thereby modulating the expression of downstream genes. 25. Previous array data employed microarray analysis to investigate the potential downstream genes of OCT4 in embryonic stem cells 19. In the course of our research, we discovered that knocking down OCT4 in mouse embryonic stem cells resulted in a significant decrease in DUSP6 expression. To verify the hypothesis that there is a positive correlation between the expression patterns of OCT4 and DUSP6 in NSCLC, the expression of these two genes was assessed in human primary lung adenocarcinoma (LUAD) specimens collected from National Cheng Kung University (NCKU) Hospital. Immunohistochemical staining revealed that tumor tissues exhibited higher levels of OCT4 and DUSP6 expression compared to normal tissues (Figure 1A). In addition, an analysis was conducted on a microarray dataset comprising 226 patients with pathological stage I-II LUAD (NCBI Gene Expression Omnibus: GSE31210) 26. As demonstrated in Figure 1B, a positive correlation was observed between the expression of POU5F1 (OCT4) and DUSP6 (Pearson's correlation coefficient, *r* = 0.1268, *p* = 0.0439). Furthermore, NSCLC cell lines with higher OCT4 expression showed greater DUSP6 levels than those with lower OCT4 expression, as confirmed by immunoblot analysis (Figure 1C). A positive correlation has been identified between OCT4 and DUSP6 expression (*r* = 0.8265, *p* = 0.0017) in these cell lines (Figure 1D), suggesting a link between DUSP6 and OCT4 expression in NSCLC. In order to study the regulatory relationship between OCT4 and DUSP6, A549 and H1299 cells were transduced with lentiviral vectors encoding OCT4 or GFP, respectively. Overexpression of OCT4 has been demonstrated to increase mRNA and protein levels of DUSP6 in A549 and H1299 cells, as examined by RT-PCR (Figure 1E, *left*), qPCR analysis (Figure 1E, *right*), and immunoblot analysis (Figure 1F), respectively. To perform OCT4 knockdown experiments, we used A549 cells due to their high transduction efficiency and high OCT4 expression. Figures 1G and 1H show that shOCT4 #5 efficiently silenced OCT4 expression, whereas shOCT4 #2 only had a minor effect. Accordingly, expression of DUSP6 was suppressed to a large extent by treatment with shOCT4 #5, but not shOCT4 #2 (Figure 1G, H). Furthermore, we investigated the effect of OCT4 overexpression on cancer cell migration and invasion. Boyden chamber assay revealed that OCT4-overexpressing A549 cells exhibited higher migratory capabilities than vector control cells (Figure 1I). Conversely, OCT4 knockdown cells have been demonstrated to attenuate cell migration and invasion (Figure 1J). Collectively, these results indicate that there is a positive correlation between OCT4 and DUSP6 expression in human primary lung adenocarcinoma specimens and NSCLC cell lines and that OCT4 overexpression enhances NSCLC cell migration and invasion.

### OCT4 transactivates DUSP6 expression by directly binding to the DUSP6 promoter in lung cancer cells

**Figure 2:**
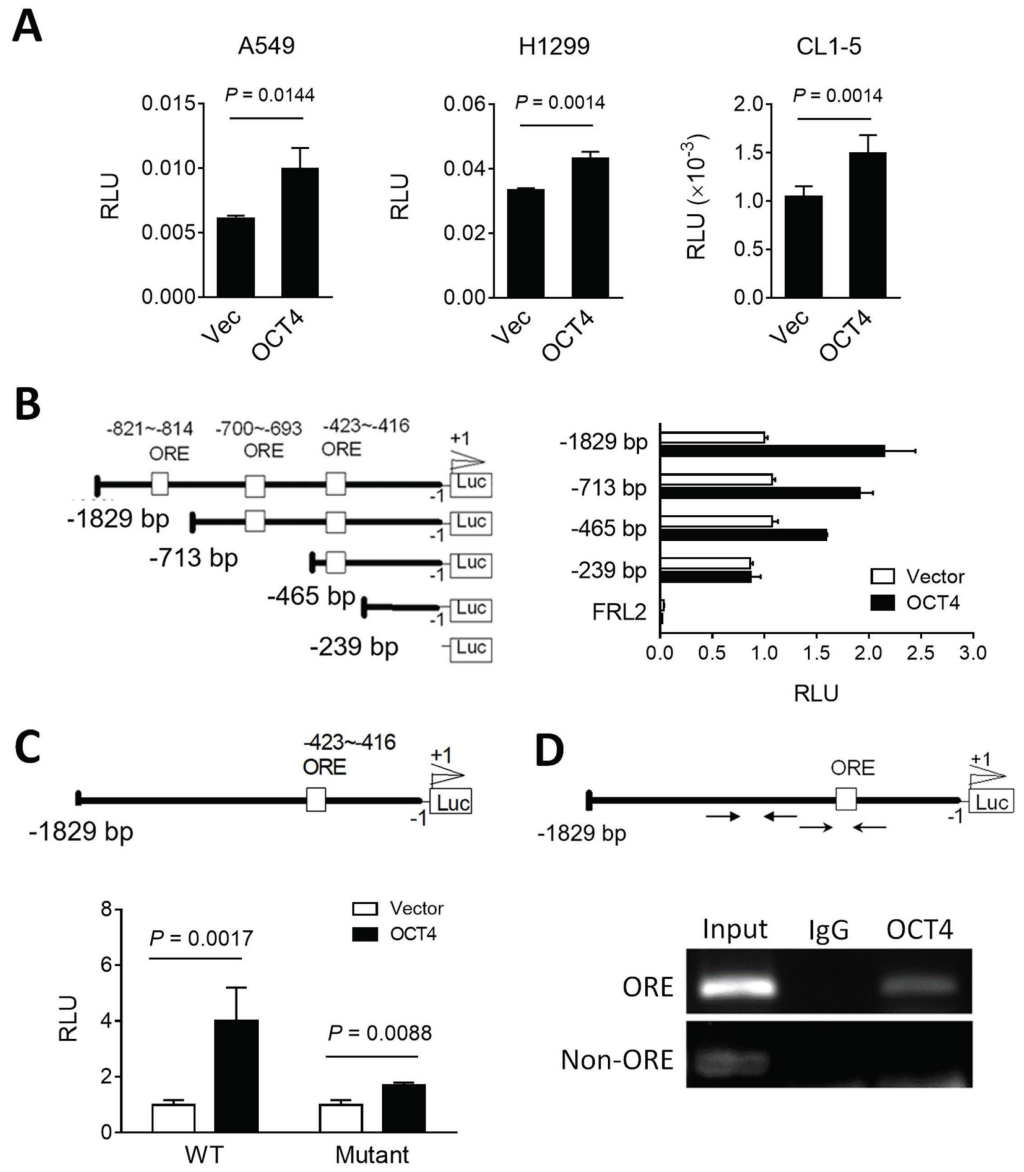
** OCT4 upregulates DUSP6 expression by transactivating the DUSP6 promoter in lung cancer cells. (A)** Reporter assay of the DUSP6 promoter in lung cancer cell lines. A549, H1299, and CL1-5 cells were co-transfected with pFRL2-hDUSP6p-Luc and pCMV-tag2B-hOCT4 (or pCMV-tag2B). **(B)** Identification of OCT4 binding sites on the human DUSP6 promoter. Schematic representation of a series of deletion constructs containing different lengths of the DUSP6 promoter. H1299 cells were co-transfected with different deletion reporter constructs of pFRL2-hDUSP6p-Luc and pCMV-tag2B-hOCT4 (or pCMV-tag2B). **(C)** A549 cells were co-transfected with pFRL2-hDUSP6p-Luc carrying a point mutation within the -423 ~ -416 ORE and pCMV-tag2B-hOCT4 (or pCMV-tag2B). Numbering is relative to the translational start site at +1. The white box indicates putative OREs. Cell lysates were harvested 48 h after transfection, and their firefly and *Renilla* luciferase activities were determined using a dual-light luciferase reporter assay system. The ratio of firefly luciferase activity to *Renilla* luciferase activity was expressed as relative light units (RLU). Data are expressed relative to the activity of the full-length promoter construct (B) or the wild-type (WT) promoter construct (C) obtained from three independent experiments (B). **(D)** ChIP assay showing direct binding of OCT4 to the ORE located at -423 to -416 within the human DUSP6 promoter. Cross-linked chromatin of H1299 cells was immunoprecipitated with mouse anti-human OCT4 antibody or normal mouse IgG in combination with protein G agarose beads, followed by PCR amplification of the DUSP6 promoter region encompassing OCT4 binding sites.

To investigate the potential of OCT4 to transactivate the DUSP6 promoter, we cloned the human DUSP6 promoter region (~2 kb) upstream of the transcription start site into a dual-luciferase reporter vector pFRL2 and co-transfected this reporter plasmid with an OCT4 expression plasmid or a control vector into A549, H1299 and CL1-5 lung cancer cell lines. The luciferase reporter assay revealed that OCT4 overexpression enhanced the DUSP6 promoter activity in these cells (Figure 2A). Analysis of the DUSP6 promoter using the Vector NTI software predicted three OCT4 binding sites, namely OCT4 response elements (OREs). To identify the key OCT4 response element (ORE) within the DUSP6 promoter, we tested a series of truncated promoter-luciferase constructs (Figure 2B, *left*) and analyzed their promoter activities (Figure 2B, *right*). OCT4 significantly activated the -1829 to 0, -713 to 0, and -465 to 0 constructs, but not the -239 to 0 construct, suggesting that the essential ORE lies between -465 and -239. Among the three predicted OREs, only the -423 to -416 site is present within this region (Figure 2B). To determine whether the predicted OCT4 response element (ORE) at positions -423 to -416 is functionally required for transcriptional activation, we introduced two point mutations within the OCT4-binding motif, altering the wild-type sequence ATGCTAAT to ATGCTAGG. This disruption of the consensus octamer motif abolished OCT4-induced activation of the DUSP6 promoter (Figure 2C), indicating that this site is essential for OCT4-mediated transcriptional regulation. Furthermore, we employed chromatin immunoprecipitation (ChIP) assays to corroborate these findings (Figure 2D). Chromatin from H1299 cells was extracted, sonicated, and immunoprecipitated using the mouse monoclonal anti-OCT4 antibody and mouse normal IgG. PCR amplification of the DUSP6 promoter region yielded 225-bp products in the OCT4 immunoprecipitates but not in the IgG control, supporting the notion that OCT4 binds to the DUSP6 promoter. Specifically, the ORE located at the -423 ~ -416 region of the DUSP6 promoter appears to be a critical binding site for OCT4, in line with the results from our luciferase promoter deletion experiments. Thus, DUSP6 is a downstream target of OCT4.

### OCT4-overexpressing A549 lung cancer cells grow faster, display higher metastatic potential, and express higher DUSP6 levels than their control counterparts in a human tumor xenograft model

**Figure 3:**
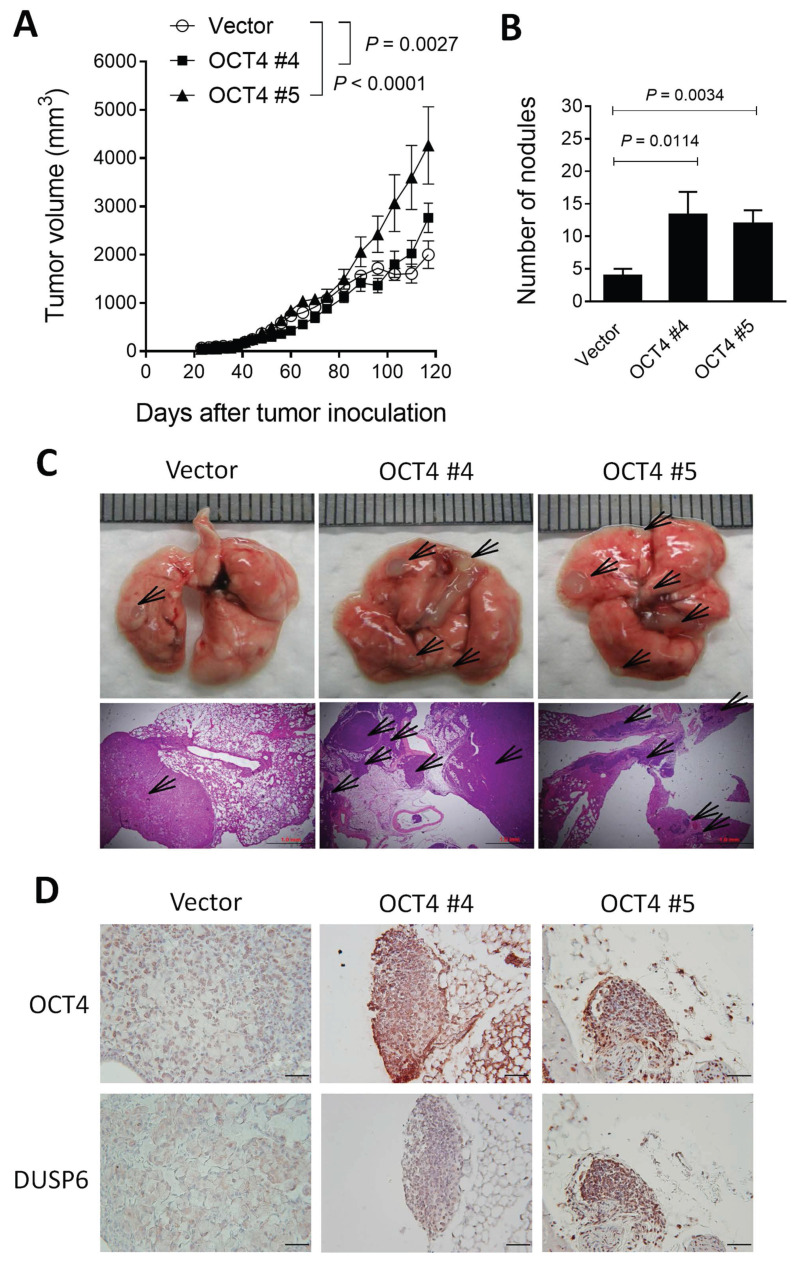
** Overexpression of OCT4 in A549 cells promotes tumor growth and metastasis and increases DUSP6 expression in mice. (A)** Tumor volumes of mice bearing OCT4-overexpressing A549 tumors. NOD/SCID mice were subcutaneously inoculated with 1 × 10^6^ cells of A549 cells overexpressing OCT4 (n=4 for OCT4 #4 and OCT4 #5) or vector control (n=4) A549 cells on day 0. Tumor volumes of the mice were measured. Tumor volumes were plotted through day 120 (primary tumor endpoint), and lungs were collected at day 156 to permit the development of spontaneous metastases in this slow‑metastasizing A549 model. **(B)** Number of metastatic nodules in the lung of individual mice. **(C)** Representative gross and histologic (hematoxylin and eosin stain, × 100 magnification, scale bar = 1.0 mm) appearances of lungs on day 156. Arrows denote tumor nodules. **(D)** Immunohistochemical detection of OCT4 and DUSP6 in paraffin-embedded lung tissue sections (× 100 magnification, scale bar = 100 μm).

To validate our *in vitro* findings, we employed a xenograft mouse model using NOD/SCID mice inoculated with OCT4-overexpressing human A549 lung cancer cells or vector control cells. A549 tumors growing subcutaneously in immunodeficient mice, such as NOD/SCID mice, can spontaneously metastasize and grow on-site in the lung tissue 27, 28. Mice bearing subcutaneous A549/OCT4 #4 or A549/OCT4 #5 tumors had larger tumor volumes compared with those bearing vector control A549 tumors (Figure 3A). In terms of tumor metastasis detected on day 156, the number of metastatic tumor nodules in the lung was significantly greater in mice bearing A549/OCT4 tumors compared with those in mice bearing control A549 tumors (Figure 3B). Figure 3C shows the gross and histological appearances of the lung tissues of tumor-bearing mice. Numbers and sizes of tumor nodules increased in mice bearing A549/OCT4 tumors compared with those in mice bearing vector control A549 tumors. Immunohistochemical staining validates higher DUSP6 expression in A549/OCT4 tumors than in control A549 tumors (Figure 3D). Taken together, these results indicate that excess expression of OCT4 in lung cancer cells promotes tumor growth and metastasis *in vivo*. Moreover, OCT4-induced DUSP6 upregulation may also play a role in tumor progression in lung cancer.

### The OCT4-DUSP6 axis enhances the migratory and invasive capability of A549 cells

**Figure 4:**
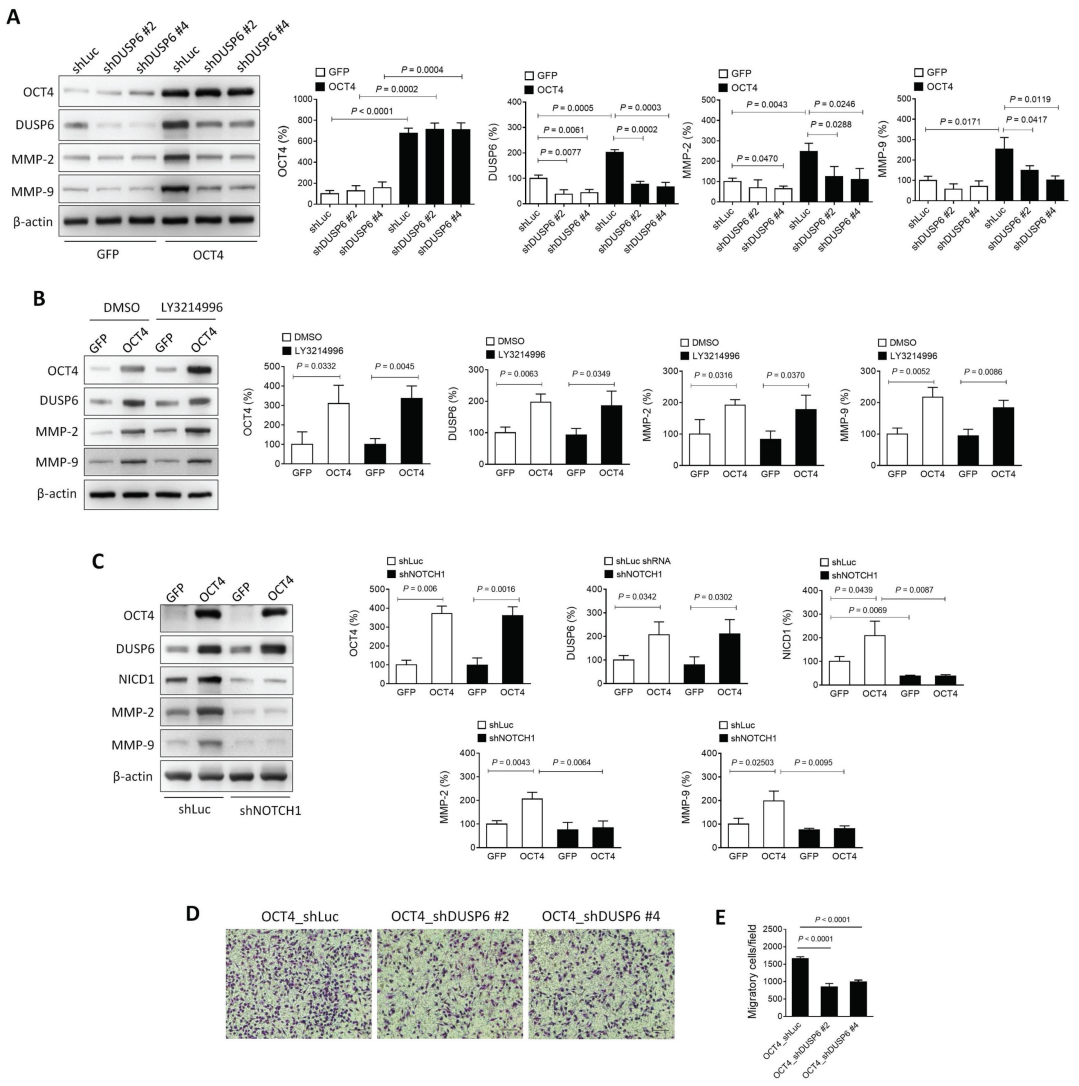
** The OCT4-DUSP6 axis promotes the migratory and invasive capability of A549 cells. (A)** OCT4-DUSP6 axis upregulates the expression of MMP2 and MMP9. A549 cells were transduced with lentiviral vectors encoding OCT4 or GFP (control), followed by puromycin selection. Subsequently, the cells were further transduced with lentiviral vectors encoding shRNA specific to DUSP6 (shDUSP6 #2 and shDUSP6 #4) or luciferase (shLuc). Detection and quantification of OCT4, DUSP6, MMP2, and MMP9 protein expression levels were examined by immunoblotting (n=3). Expression of β-actin served as the loading control.** (B)** The OCT4-DUSP6 axis regulates MMP2 and MMP9 expression independently of the inhibition of ERK1/2-MAPK activity. OCT4 or GFP-transduced A549 cells were treated with DMSO or ERK inhibitor (LY3214996, 2μM) for 48 h. Total protein extracts were detected and quantified for OCT4, DUSP6, NICD, MMP2, and MMP9 expression (n=3).** (C)** The OCT4-DUSP6 axis regulates MMP2 and MMP9 expression through the Notch pathway. A549 cells were transduced with lentiviral vectors encoding OCT4 or GFP, followed by further transduction with lentiviral vectors encoding shRNA specific to NOTCH (shNOTCH) or luciferase (shLuc). Detection and quantification of OCT4, DUSP6, NICD, MMP2, and MMP9 protein expression levels were examined by immunoblotting (n=3). **(D)** Migratory and invasive capabilities of DUSP6-knockdown and Luc control A549/OCT4 cells. Cells that migrated through the membrane to the lower surface in the Boyden chamber were stained with Giemsa solution and visualized by light microscopy (× 200 magnification, scale bar = 200 μm) (B) and quantified (C). Migratory cells in three fields in each membrane were counted. All quantification values and error bars shown were mean ± SEM, and P-values less than 0.05 were shown in the quantitative charts. NICD, Notch intracellular domain; SEM, standard error of the mean.

Matrix metalloproteinases (MMPs), particularly MMP-2 and MMP-9, are critical mediators of cancer cell migration and metastasis 29, 30. In NSCLC, these MMPs are frequently upregulated, and their inhibition has been shown to reduce tumor invasion and metastasis 31, 32. OCT4 is known to elevate MMP-2 and MMP-9 expression, thereby promoting metastasis 18. To investigate whether DUSP6 contributes to this regulation, two DUSP6-knockdown clones (shDUSP6 #2 and #4) and control cells (shLuc) were generated in OCT4-overexpressing A549 cells. Immunoblotting revealed that the OCT4-induced upregulation of MMP-2 and MMP-9 was abolished in cells with reduced DUSP6 expression (Figure 4A), suggesting that DUSP6 mediates the OCT4-driven expression of MMP-2 and MMP-9.

DUSP6, a MAPK phosphatase, typically regulates ERK1/2 through negative feedback 4. However, treatment with the ERK inhibitor LY3214996 did not suppress the OCT4-induced expression of DUSP6, MMP-2, or MMP-9 (Figure 4B), indicating that the OCT4-DUSP6 axis upregulates MMP-2 and MMP-9 independently of the MAPK/ERK pathway. In addition to its MAPK-related role, DUSP6 has been reported to act as a phosphatase for Notch1, thereby regulating Notch1 transmembrane/intracellular region (NTM) stability and Notch1 intracellular domain (NICD) transcriptional activity 33. Since the Notch pathway also influences MMP-2 and MMP-9 expression in NSCLC 34-37, its involvement in OCT4-DUSP6 signaling was examined. Immunoblot analysis demonstrated that knockdown of Notch1 (shNOTCH1) in A549/OCT4 cells abolished the OCT4-induced upregulation of DUSP6, MMP-2, and MMP-9 (Figure 4C), implicating that the OCT4-DUSP6 axis modulates MMP-2 and MMP-9 expression via the Notch pathway. Moreover, Boyden chamber assays demonstrated that reducing DUSP6 levels led to a twofold decrease in cell migration in OCT4-overexpressing cells (Figure 4D and 4E). Taken together, these findings suggest that the OCT4-DUSP6 axis promotes cancer cell migration and invasion by upregulating MMP-2 and MMP-9 through a Notch-dependent, MAPK-independent mechanism, highlighting its potential role in NSCLC progression.

### Knockdown of DUSP6 in OCT4-overexpressing A549 lung cancer cells decreases tumor growth and metastasis in a human tumor xenograft model

**Figure 5:**
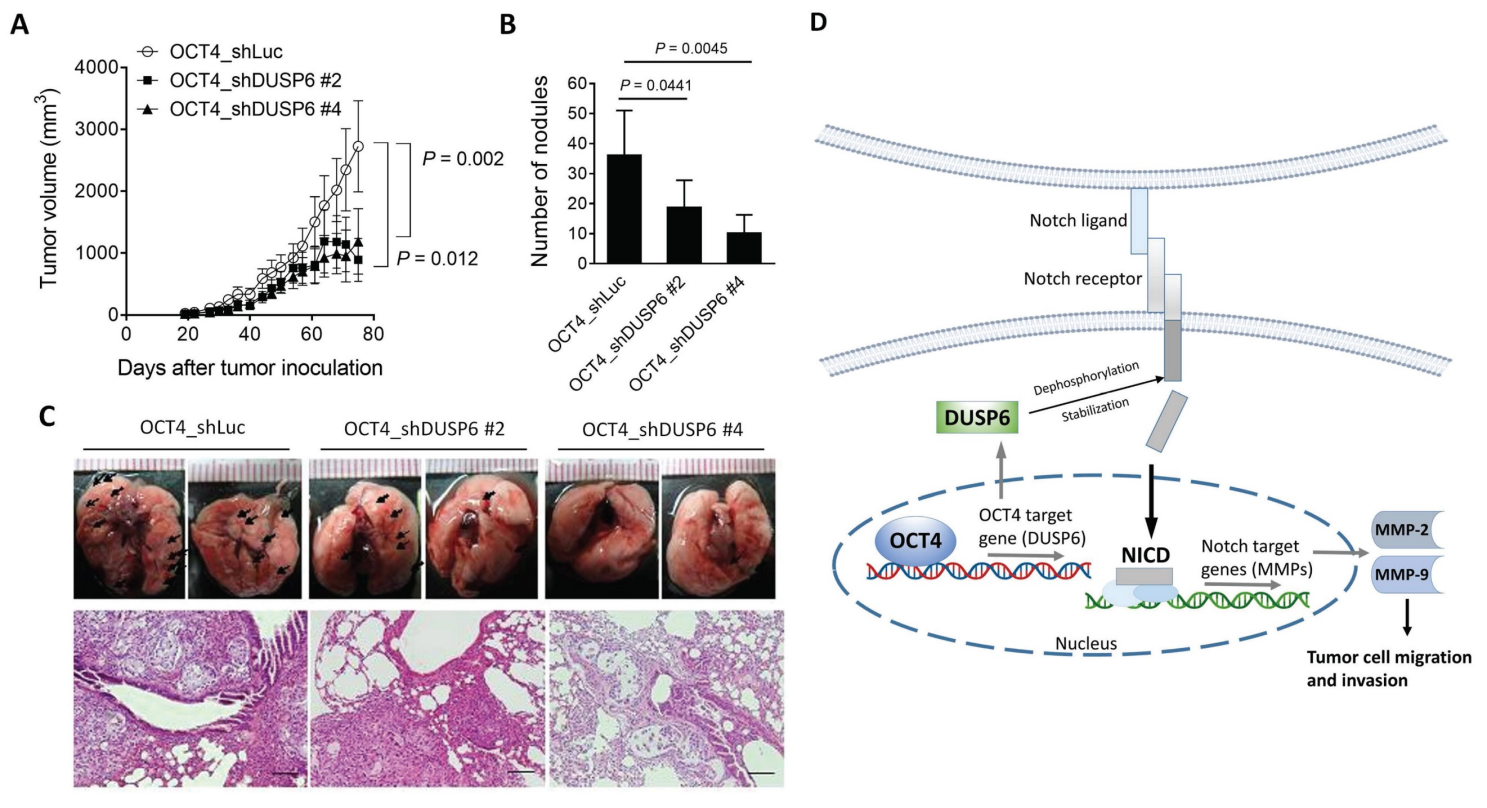
** Knockdown of DUSP6 in OCT4-overexpressing A549 cells decreases tumor growth and metastasis in mice. (A)** Tumor volumes of mice bearing DUSP6-knockdown or vector control A549/OCT4 tumors. NOD/SCID mice were subcutaneously inoculated with 1 × 10^6^ cells of DUSP6-knockdown (n=6 for OCT4_shDUSP6#2; n=7 for OCT4_shDUSP6#4) or vector control A549/OCT4 cells (n=7 for OCT4_shLuc). Tumor volumes of the mice were measured. **(B)** Number of metastatic nodules in the lung of individual mice. **(C)** Representative gross and histologic (hematoxylin and eosin stain, × 200 magnification, scale bar = 1.0 mm) appearances of lungs on day 86. Arrows denote tumor nodules. (**D**) A schematic representation of the OCT4-DUSP6 axis involved in lung cancer progression. OCT4 overexpression in NSCLC cells upregulates DUSP6. DUSP6 has been shown to function as a phosphatase for Notch1, thereby regulating Notch1 transmembrane/intracellular region (NTM) stability and Notch1 intracellular domain (NICD) transcriptional activity, resulting in driving tumor aggressiveness through MMP2 and MMP9 upregulation. The pathways in gray color have been reported previously.

To further confirm the relevance of *in vitro* DUSP6 knockdown results (Figure 4D, 4E), we inoculated DUSP6-knockdown or vector control OCT4-overexpressing A549 cells into NOD/SCID mice to investigate whether knockdown of DUSP6 expression could attenuate tumor growth and metastasis in A549/OCT4 tumor-bearing mice. Figure 5A shows that knockdown of DUSP6 expression significantly reduced subcutaneous tumor growth in tumor-bearing mice. Quantification of lung tumor nodules revealed that mice bearing A549/OCT4_shDUSP6 tumors had a lower potential to metastasize to the lung compared to those bearing A549/OCT4_shLuc tumors (Figure 5B). Gross and histological examinations of lung tissues revealed that the numbers and sizes of tumor nodules appeared to decrease in mice bearing DUSP6-knockdown A549/OCT4 tumors, in particular shDUSP6 #4-knockdown tumors, compared with those in mice bearing vector control A549/OCT4 tumors (Figure 5C). Collectively, these results indicate that knockdown of DUSP6 expression in OCT4-overexpressing lung cancer cells reduces tumor growth and metastasis in mice bearing A549 human lung tumor xenografts. These *in vivo* results underscore the pivotal role of DUSP6 in contributing to OCT4-induced tumor growth and distant metastasis.

## Discussion

DUSP6, a member of the dual specificity protein phosphatases family, acts as a negative ERK1/2 regulator by dephosphorylating tyrosine and serine/threonine residues. Its role in promoting tumorigenesis across various cancers is increasingly recognized. DUSP6 overexpression not only enhances *in vitro* cancer cell colony formation and migration but also accelerates tumor growth *in vivo*. Notably, its overexpression is evident in human breast cancer 38, glioblastoma 10, papillary thyroid cancer 8, 9, and NSCLC 7, where it is identified as a key factor in predicting relapse-free and overall survival. In the present study, we show that DUSP6 is overexpressed in human NSCLC tissues as compared to adjacent normal lung tissues and is expressed at different levels in various human lung cancer cell lines, including NSCLC cells and H661 large cell lung cancer cells.

The clinical relevance of OCT4 and DUSP6 in cancer recurrence has been documented. We have previously analyzed RNA expression profiles in human lung adenocarcinoma in the Oncomine database (accession no. GSE31210) 26. Kaplan-Meier analysis revealed that lung adenocarcinoma patients with high OCT4 expression (n = 336) had shorter relapse-free survival than those with low expression (n = 646) (p < 0.01) 39. In bladder cancer, we have examined the expression levels of OCT4 in clinical specimens of superficial high-grade (stages T1-2) bladder transitional cell carcinoma of 110 patients. Our results demonstrate that patients with OCT4 high-expressing tumors (n = 28) had significantly shorter recurrence-free intervals (median = 13 months) than those with OCT4 low-expressing tumors (n = 82; median = 34.5 months) (p < 0.0001) 40. In addition, DUSP6 was reported to be upregulated in early lung cancer lesions with activating EGFR or RAS mutations 41. Moreover, DUSP6 is part of a five-gene signature that predicts relapse-free and overall survival in patients with NSCLC 7.

Cancer cells acquire and maintain CSC characteristics in response to harsh tumor microenvironment conditions. A mathematical model was proposed that these emerging adaptive behaviors in cancer might be driven by harsh selective forces in the tumor microenvironments 42. It has been demonstrated that DUSP6 promotes resistance to serum starvation and inhibits apoptosis via Akt phosphorylation, potentially contributing to the acquisition of a CSC phenotype 43. These results suggest that DUSP6 has a potential value as a biomarker of CSCs and as a target of therapies designed to eliminate CSCs.

While the expression of DUSP6 in multiple cancer types has been documented, its regulatory mechanisms remain elusive. The role of p53 as a transactivator of DUSP6 in colorectal HCT116 cells has been established, implicating that p53 plays a role in DUSP6 mRNA transcription 44. However, expression of DUSP6 in p53 knockout HCT116 cells suggests additional regulatory pathways. It was shown that expression of MKP3 (DUSP6) is increased in cisplatin-resistant NSCLC cells and lung tumor xenografts 45. Knockdown of DUSP6 increases levels of insulin growth factor binding protein 7 (IGFBP7), a secreted protein regarded as a tumor suppressor, indicating that DUSP6 regulates IGFBP7. Furthermore, DUSP6 increases resistance to tamoxifen treatment in breast cancer 46. These findings suggest a novel molecular mechanism of DUSP6 in drug-resistant lung cancer.

During hypoxia, a hallmark of tumor growth in the tumor environment, hypoxia-induced factor (HIF)-1α upregulates DUSP6 mRNA expression 47, showing that post-transcriptional regulation is a key process in the control of DUSP6 expression. OCT4 is a marker of CSCs 48. In the present study, we identify OCT4 as a novel DUSP6 regulator. OCT4, typically upregulated by HIF-2α under hypoxic conditions 49, is overexpressed in NSCLC tissues and cell lines, which correlates with DUSP6 expression. Furthermore, overexpression of OCT4 in A549 and H1299 NSCLC cells increases mRNA and protein levels of DUSP6, whereas knockdown of OCT4 reduces DUSP6 expression. Importantly, luciferase reporter and ChIP assays revealed that OCT4 upregulates DUSP6 expression at the transcriptional level by binding directly to the ORE within the DUSP6 promoter. This novel finding demonstrates that DUSP6 is a downstream target of OCT4. OCT4 upregulation has been observed in various cancers, including NSCLC 15, 16, 22, 50, melanoma 51, and glioblastoma 14. Its expression correlates with advanced cancer progression and poorer clinical outcomes. In the current study, overexpression of OCT4 enhances the migratory capability of A549 cells *in vitro*, contributing to cancer metastasis *in vivo*. This effect may be partially mediated by metalloproteinases (MMPs, such as MMP-2 and MMP-9), which are increased by DUSP6 upregulation since reduced MMPs have been found in DUSP6-deficient polymorphonuclear neutrophils (PMNs) even without stimulation by phorbol esters 52. We also show that suppression of DUSP6 expression reverses the enhanced motility and growth of OCT4-overexpressing A549 cells both *in vitro* and *in vivo*, highlighting a new pathway for the role of OCT4 in promoting lung cancer growth and metastasis.

(E/Z)-BCI hydrochloride (BCI) is a small molecule inhibitor of DUSP6 with anti-inflammatory activities 53. Inhibition of DUSP6 by BCI suppresses proliferation, migration, and invasion and induces apoptosis in gastric cancer cells 54. Furthermore, inhibition of DUSP6 sensitizes gastric cancer cells to cisplatin-induced cell death and apoptosis *in vitro* and *in vivo*. Thus, BCI, in combination with cisplatin, represents a novel therapeutic strategy for the treatment of gastric cancer 54. In the present study, our results show that the knockdown of DUSP6 alleviates not only primary tumor growth but also pulmonary metastasis in NOD/SCID mice bearing OCT4-overexpressing A549 lung tumor xenografts. Therefore, pharmacological inhibitors of DUSP6, such as BCI, as well as modulation of OCT4 and DUSP6 expression, may be further explored for treating OCT4-overexpressing lung cancer. Future research may also focus on studying the detailed molecular mechanism of BCI underlying DUSP6 inhibition. Clinical applications of BCI in overcoming drug resistance and metastasis for the treatment of lung cancer with OCT4 and DUSP6 upregulation are warranted.

The role of DUSP6 in cancer is complex and somewhat contradictory. While it functions as a phosphatase inactivating ERK1/2, its impacts on cancer cell proliferation, migration, and apoptosis may be different. Some lines of evidence suggest a tumor-suppressing role for DUSP6, as observed in aerodigestive tract cancers, where its downregulation correlates with tumor formation and poor survival 55. Conversely, other studies reported that DUSP6 overexpression fosters colony formation and migration in breast cancer 38 and glioblastoma cells 10, highlighting the nuanced and context-dependent nature of the role of DUSP6 in cancer. DUSP6 is upregulated in EGFR- or KRAS-mutant lung adenocarcinoma cells, potentially protecting cells with mutations in the RAS signaling pathway, a proposal supported by experiments with DUSP6-specific siRNAs and pharmacological inhibitors 41. Targeting DUSP6 or other negative regulators might offer a treatment strategy for certain cancers by inducing the toxic effects of RAS-mediated signaling. Future studies are warranted to explore the broader genetic and signaling networks interacting with the OCT4-DUSP6 pathway to provide a more comprehensive understanding of the progression of NSCLC. Furthermore, in-depth exploration of the therapeutic potential of targeting the OCT4-DUSP6 axis is valuable for translational medical research.

Our study reveals a novel mechanism by which OCT4 promotes the progression of NSCLC through the upregulation of DUSP6, leading to enhanced migratory and invasive capabilities of cancer cells. Interestingly, while DUSP6 is traditionally associated with negative regulation of the MAPK/ERK pathway, our data demonstrate that the OCT4-DUSP6 axis induces MMP-2 and MMP-9 expression independently of this canonical pathway. Instead, we identified the Notch signaling pathway as a key mediator in this regulatory process. Notably, knockdown of Notch1 abolished OCT4-induced expression of DUSP6, MMP-2, and MMP-9, highlighting a previously unrecognized functional link between OCT4, DUSP6, and Notch signaling. This finding suggests that DUSP6 may serve as a critical molecular bridge between OCT4 and Notch pathways, both of which are known to play central roles in CSCs regulation (Figure 5D). Our results provide new insights into the molecular mechanisms underlying NSCLC metastasis and suggest that targeting the OCT4-DUSP6-Notch signaling axis may hold therapeutic potential. However, further studies are warranted to fully elucidate the detailed molecular interactions and validate this axis in in vivo models and clinical samples.

In the present study, we demonstrate that OCT4 exacerbates tumor growth and metastasis in lung cancer by transactivating DUSP6 expression. This transactivation, facilitated by direct binding of OCT4 to the DUSP6 promoter, is crucial for the growth and migration of cancer cells promoted by OCT4. Overexpression of both OCT4 and DUSP6 in NSCLC tissues and cell lines, coupled with their positive correlation, suggests their potential as prognostic markers. Moreover, targeting the OCT4-DUSP6 pathway may offer a new therapeutic avenue for NSCLC, as evidenced by reduced tumor growth and lung tumor nodules in mice bearing OCT4-overexpressing A549 lung tumors after silencing DUSP6 expression.
